# No good vaccination quality without good control: the positive impact of a hatchery vaccination service program

**DOI:** 10.1016/j.psj.2020.03.017

**Published:** 2020-04-15

**Authors:** Giovanni Franzo, Wessel Swart, Wiliam Boyer, Daniela Pasotto, Gema Ramon, Kostas Koutoulis, Mattia Cecchinato

**Affiliations:** ∗ Department of Animal Medicine, Production and Health (MAPS), University of Padua, 35020 Legnaro (PD), Italy; † Ceva Santé Animale, 33500 Libourne, France; ‡ Department of Poultry Diseases, Faculty of Veterinary Medicine, University of Thessaly, Karditsa, Greece

**Keywords:** vaccination, hatchery, performance evaluation, questionnaire

## Abstract

Vaccination is currently one of the most relevant control strategies in poultry production to reduce infectious disease–induced economic losses and decrease antimicrobial use. Besides intrinsic vaccine efficacy, a proper administration is fundamental to achieve an adequate coverage and protection. Hatchery vaccination is becoming the standard approach for routine vaccination because of administration easiness, the possibility to standardize and optimize the overall process, and the lower impact on animal welfare compared with different types of on-farm vaccination. However, a continuous maintenance, refinement, and training of the personnel are the key to success. In the present work, the effect of longitudinal hatchery audits, performed using a standardized, expert-developed questionnaire was evaluated in 169 hatcheries, located in 11 European countries, over a period of more than 4 yr. A dedicated tablet-based application was implemented for data collection, storage, and analysis, and the obtained scores were used in the evaluation, reporting to the hatchery management and improvement of critical points. A positive significant association was demonstrated between the variation in global and process-specific hatchery scores and the number of performed audits. Similarly, when the longitudinal nature of the data (i.e., multiple visits) was accounted for using linear mixed models, including the hatchery and country as random factors, a significant trend in performance improvement was observed visit after visit, although with certain differences based on the specific score and country. The present study demonstrates the benefits of an objective evaluation of hatchery performances through a standardized questionnaire, followed by the discussion on the major required actions. The widespread application of this approach should lead to a significant improvement in vaccine administration performances, with direct consequences on infectious disease occurrence and animal production performances, and indirectly on therapeutic and control-related costs.

## Introduction

Infectious diseases represent a major cause of economic loss for the poultry industry. Besides direct costs owing to increased mortality and decreased productive performances, several indirect additional costs can be identified. Constraints to animal management and handling, implementation of effective biosecurity measures, increased susceptibility to other infectious and non-infectious diseases, and treatment-related cost and condemnation can severely affect the farm profitability (McLeod et al., 2016).

The use of antimicrobials, either against primary pathogens or opportunistic ones (typically benefiting from primary viral infections) is nowadays strongly discouraged and limited by national and international legislation and health institutions, especially within the European Union borders (Murphy et al., 2017).

Moreover, the crescent trend for “antibiotic-free” products is leading to a decreased economic value of the antimicrobial-treated livestock and their processed products (Goddard et al., 2017, Ancillotti et al., 2018).

Effective vaccines are a pivotal tool in this framework, as they are preventing or decreasing animal susceptibility to infections and related consequences. In addition to vaccine intrinsic efficacy, a proper administration and high coverage severely influence the overall protection at flock level (de Wit et al., 2010, Dortmans et al., 2012). Several studies have demonstrated how poor vaccine management can significantly affect the efficacy even of vaccines proven to be highly protective in experimental conditions (de Wit et al., 2010).

Improper vaccination can therefore allow for pathogen circulation, exposing animals to infection and disease, and decrease productivity (Franzo et al., 2016a). Moreover, field pathogen circulation in vaccinated populations has been associated to an increased risk of vaccine-induced immune escape or vaccine field strain recombination (Li et al., 2009, Lee et al., 2012, Franzo et al., 2016b, Moreno et al., 2017), leading to the emergence of new strains whose biological features are hardly predictable. When vaccination coverage is low, prolonged rolling reaction could favor episodes of reversion to virulence, leading to vaccine-induced clinical outbreaks (Nielsen et al., 2001, Catelli et al., 2006, Cecchinato et al., 2014) or opportunistic pathogen infections (Franzo et al., 2014), thus enhancing a certain skepticism against this fundamental control tool.

Hatchery vaccination displays several advantages, by allowing for more standardized administration protocols and a higher control of the whole process by specifically trained personnel and well-operated vaccination technologies (Abdul-Cader et al., 2018). For these reasons, a crescent number of vaccines, against different diseases, have been licensed to administer either before hatch or immediately after hatch, including both traditional and new technology vaccines (Comte, 2013, Abdul-Cader et al., 2018).

In this context, the optimization of hatchery activity is of pivotal relevance for the health status and profitability of the downstream rearing phases.

The remarkable improvement and diffusion of informatics tools have encouraged the development of user-friendly applications to monitor different production phases, processes, and diagnostics.

In the present study, the usefulness of a hatchery vaccination service program implemented in several European countries was evaluated. This hatchery vaccination program, called the Ceva Hatchery Immunization Control Keys (**CHICK**) program, involves specialist teams visiting the hatcheries in more than 40 countries worldwide on a frequent basis, to audit the vaccination process, to secure vaccination technology maintenance, to train the hatchery personnel, and to improve their performance. During the audits, the different parameters are scored, using a tablet-based application, and the results are stored in a dedicated database to be reported to hatchery management. Particularly, the effect of multiple audits on overall hatchery performances and specific processes was assessed from the data obtained during European hatchery monitoring activity.

## Material and methods

### Data Collection

In the context of a routine hatchery vaccination service program, called the CHICK program, consisting of audits, trainings, equipment maintenance, and advices for improvements, a dedicated questionnaire was filled and recorded on an application (CHICK app) by the members of an internal specialist team formed by Ceva employees. They are qualified professionals whose activity to control good hatchery vaccination practices is structured by a Quality Code of Practice (Ref. CT814 V6.04/10/19) for which compliance is testified by an independent external auditing company (Bureau Veritas Group). Particularly, the considered vaccination protocols were spray and day-old subcutaneous vaccination.

The questionnaire was developed by experts with a strong knowledge of the situation in the field, who defined the parameters to be evaluated and their relative weight. The structure of the questionnaire is organized in chapters where different types of vaccination and vaccine can be selected and investigated. Within each chapter, different aspects of the quality of vaccine storage, vaccine preparation, vaccination performances, equipment care and maintenance, training of the technicians, and chick quality can be scored. The scoring is mainly carried out by closed questions (yes/no, preselected answers) and by some open questions for the reporting. Based on the results, several partial scores (i.e., dealing with particular issues: vaccine preparation, spray equipment maintenance, spray quality, injection equipment maintenance, and injection quality) and a global score of the hatchery are calculated.

**Figure 1:**
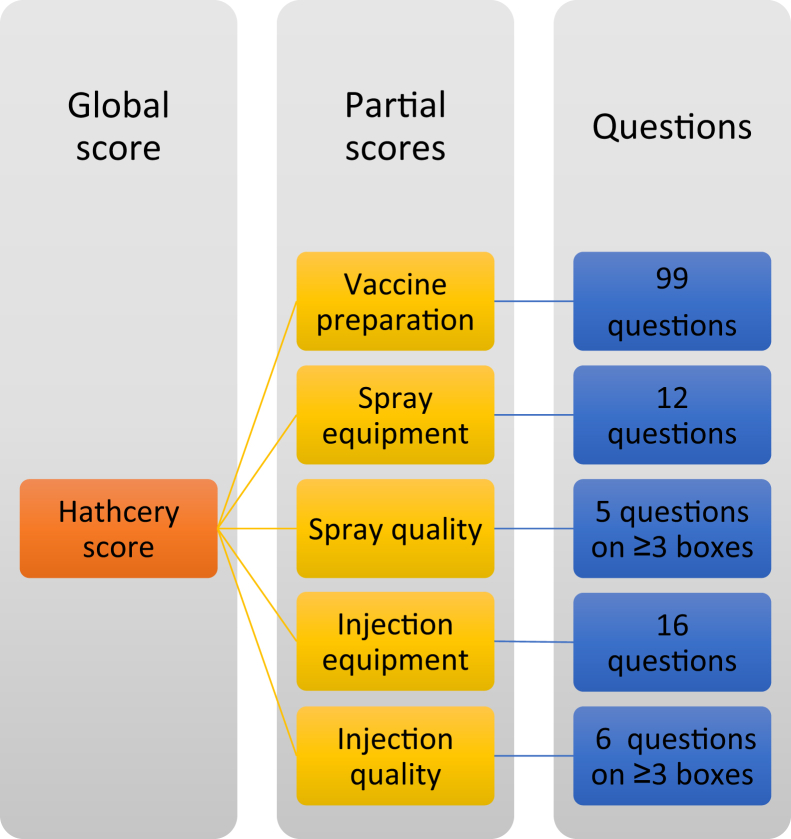
Hierarchical structure of the questionnaire.

The hierarchical structure of the scoring system is summarized in Figure 1. The CHICK program is a registered CEVA product, and the precise implementation cannot be disclosed; however, interested readers can refer to the authors to obtain more detailed information on its underlying structure and principles. The data are stored in the database for further analysis, internal and external benchmarking, and future reporting. Particularly, the results, including comments and pictures, are reported in a graphic way to the hatchery management, comparing them with past performances. The hatchery status is discussed with the responsible personnel and the managers, potential ameliorating actions are suggested and the results followed up in the next visit.

### Statistical Analysis

Before analysis, all hatcheries where less than 3 visits had been performed were excluded because the main study aim was to assess the benefits of a prolonged hatchery monitoring.

For each hatchery, the total number of visits was counted, and the difference between the first and last visit score was recorded. In addition, the time interval (in mo) between these 2 events was used to calculate the average visit frequency for each hatchery.

The relationship between each score improvement and total visit number or visit frequency was independently assessed through linear regression.

Because first and last measure alone could be poorly representatives of the overall pattern, more complex, linear hierarchical mixed models were developed for each considered score to account for the longitudinal nature of data. More in detail, the score obtained in each visit was used as an outcome variable, whereas the visit progressive number (i.e., first visit, second visit, …, n-visit) was selected as an ordinal predictor variable (fixed effect). To model the data nature more accurately, additional parameters were added in a stepwise fashion:

1) Hatchery was added as random effect allowing an independent regression intercept for each one.
2) An independent slope was allowed for each hatchery.
3) The hierarchical structure (i.e., hatchery nested within country) was added.
4) The correlation between measurements (visits) was accounted for and different autoregressive models were evaluated.

Normality and homoscedasticity of residuals were graphically inspected and tested by Shapiro–Wilk and Breusch–Pagan test for all considered models. All models were fitted using the *nlme* (Pinheiro et al., 2013) library in R 3.4.4, and the statistical significance of each model improvement over simpler ones was assessed by likelihood-ratio test.

The statistical significance level was set to *P*-value<0.05 for all mentioned tests.

## Results

### Data Set

A total of 1,678 visits were performed over a more than 4-yr period (January 2015–April 2019) in 169 hatchery, located in 11 European countries. The number of visits per hatchery ranged between 1 and 57, with an average value of 9.92. However, hatcheries where less than 3 visits had been performed were excluded from further analysis (included hatcheries = 115; average visit number = 13.96).

### Improvement Between First and Last Visit

**Figure 2:**
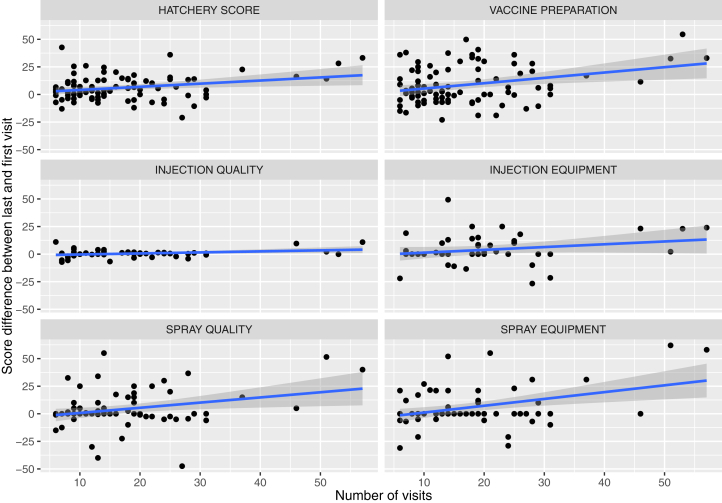
Scatter plot reporting the variation in global and partial scores between last and first visit plotted against the number of occurred visits. A regression line and relative 95 confidence interval (shaded area) have been superimposed.

Overall, the global hatchery score improved as the total number of visits performed (b = 0.284; *P*-value = 0.011) (Table 1). Similar results were obtained for partial scores: injection quality (b = 0.093; *P*-value = 0.023), spray equipment maintenance (b = 0.618; *P*-value 0.001), spray quality (b = 0.468; *P*-value 0.014), and vaccine preparation (b = 0.485; *P*-value 0.004). Nevertheless, no association could be detected with the injection equipment maintenance (b = 0.254; *P*-value = 0.129) (Table 1 and Figure 2).

**Table 1:** Summary of linear regressions and mixed effect linear regressions parameters fitted for different hatchery scores; Statistical significance is also reported.

Model	Explanatory variable	Score	B^1^	SE	T-value	*P*-value
Linear model	Visit number	Global score	0.284	0.109	2.591	0.011
		Vaccine preparation score	0.484	0.164	2.952	0.004
		Spray equipment score	0.617	0.188	3.272	0.001
		Spray quality score	0.468	0.185	2.528	0.014
		Injection equipment score	0.253	0.164	1.546	0.129
		Injection quality score	0.0934	0.040	2.329	0.024
	Visit frequency	Global score	1.325	7.714	0.172	0.864
		Vaccine preparation score	3.075	11.356	0.271	0.787
		Spray equipment score	−3.927	11.296	−0.348	0.729
		Spray quality score	−4.710	11.599	−0.406	0.686
		Injection equipment score	−11.863	12.357	−0.960	0.343
		Injection quality score	2.088	3.054	0.684	0.498
Mixed model	Visit number	Global score	0.2661	0.113	2.353	0.018
		Vaccine preparation score	0.4468	0.149	2.985	0.002
		Spray equipment score	0.2548	0.070	3.626	>0.001
		Spray quality score	0.363	0.098	3.696	>0.001
		Injection equipment score	0.122	0.123	0.987	0.323
		Injection quality score	0.021	0.024	0.862	0.388

1 Regression coefficient.

No association was demonstrated between average monthly visit frequency and global or partial hatchery score improvement (Table 1). Comparable results were obtained when analysis were repeated excluding hatcheries featured by extreme visit frequency (>2 and > 1 average visits per month) (Data not shown).

**Figure 3:**
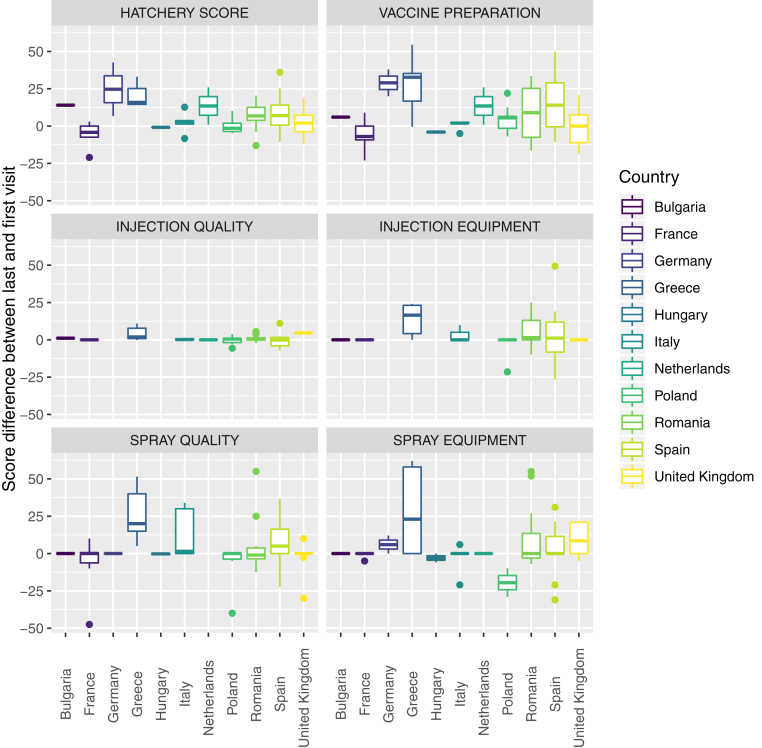
Boxplots representing the variation in Global and partial scores between the last and first visit to the heathery. The countries included in the study have been color-coded.

A clear difference in improvement magnitude between the first and last visit could be observed among the considered countries (Figure 3).

### Mixed Model Analysis

**Figure 4:**
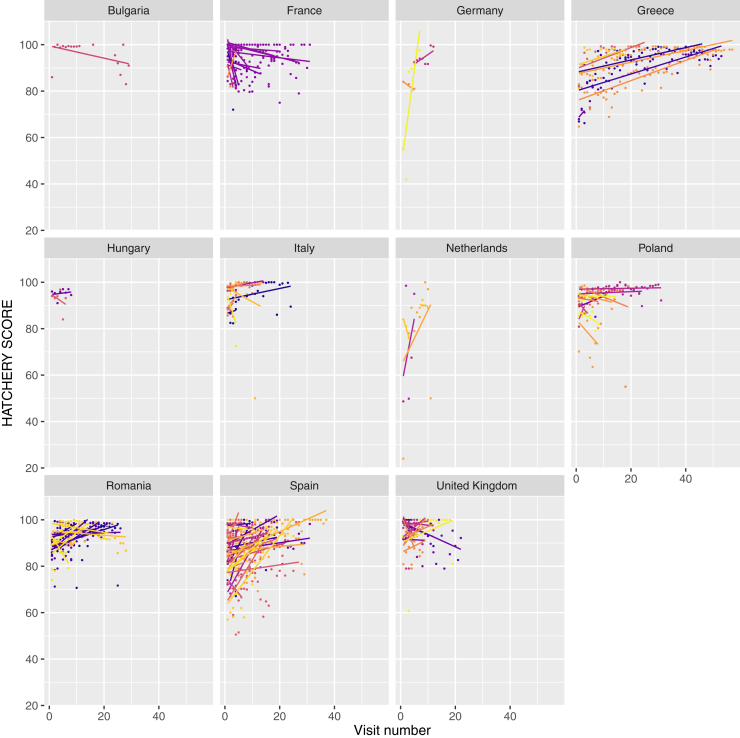
Scatter-plot reporting the global score against the visit events, chronologically ordered. Each facet describes a different country. A lineal model has been fitted for each hatchery (visit-specific scores and the regression lines have been color coded). Only the global score results are provided for graphical reasons.

Overall, the best mixed model included visit events (longitudinally performed questionnaires) as fixed effect and hatchery, nested within country, as random effect. Particularly, a model allowing for independent intercepts and slopes for each hatchery guaranteed a significant model improvement compared with simpler ones (Figure 4). Finally, when temporal correlation structure among observations was evaluated, an autocorrelation structure of order 1 with visit as covariate and hatchery, nested within country, as grouping factor was selected.

The effect of audits on the global score was positive and statistically significant (b = 0.266; *P*-value = 0.018) because an average 0.266% improvement in the global score was expected for each additional visit. Considering partial scores, a positive and significant association was also found between visit and vaccine preparation, spray equipment maintenance, and spray quality scores, whereas no association was detected with injection equipment maintenance and injection quality scores (Table 1).

## Discussion

Vaccination represents a matchless tool against many livestock infectious diseases, which often allows effectively controlling clinical signs occurrence and severity (Lombard et al., 2007). Although the infection itself is rarely prevented, the higher resistance to infection coupled with the lower pathogen replication and shedding in immunized animals can significantly reduce the basic reproduction number (R_0_) (de Wit et al., 1998), thus decreasing outbreak size and spread potential. Nevertheless, a proper vaccine administration is pivotal to obtain an effective animal response and protection (de Wit et al., 2010, Franzo et al., 2016a). Although this issue could seem obvious, it represents a major challenge for many livestocks and poultry in particular, where the huge population size hinders individual vaccination in favor of the mass one (de Wit et al., 2010, Dortmans et al., 2012). Although practical, this strategy implies a loss of control that can be detrimental to animal coverage and protection, potentially leading to vaccine reactions, rolling reactions, reversion to virulence, and so on (Cecchinato et al., 2014, Franzo et al., 2016a). For these reasons, hatchery vaccination has gained a crescent favorbecause it potentially allows for a better management and standardization of the whole procedure for both mass and individual vaccination. However, a constant monitoring and refinement of operational procedures are mandatory.

In the present study, the effect of longitudinal audits performed on vaccination performances of more than 150 European hatcheries has been evaluated, thanks to the implementation of a practical and standardized scoring system whose results could be stored and analyzed in a comparative way.

Overall, a positive relationship could be observed between the visit number and global score, which was further strengthened when the longitudinal nature of the data was accounted for. Therefore, an objective and constant measurement of the hatchery performance was proven to be particularly useful to discuss the main action points with hatchery personnels and evaluate the evolution over time by comparing the results of several visits. Moreover, the development of a tablet-based software app and related data storage system allowed for an easy data collection and analysis, providing graphical evidences and summary statistics that were useful to contextualize the particular audit within the historical record of the hatchery and, if necessary, to compare it with other productive scenario (i.e., same country, hatcheries with similar features, and so on).

On the other hand, an association with visit frequency could not be detected. Although this could appear counterintuitive, it must be considered that many procedures and personnel training require time to be implemented, minimizing the benefits of frequent, close audits.

Accordingly, when partial scores were considered, a positive relationship could be detected between longitudinal monitoring and scores associated to vaccine preparation and spray vaccination administration but not with vaccine injection–related parameters. While the hatchery spray vaccination equipment used by the hatcheries subject to this research has all been introduced relatively recently and therefor requires frequent calibration and optimization steps, the vaccine injection equipment has been installed already for a longer period. Therefore, only minimal improvements in routinely applied procedures can be expected. These results suggest the practical relevance of the developed questionnaire to detect the critical points and focus the efforts in solving them, without dissipate resources in less beneficial actions.

Interestingly, statistical model comparison revealed that the inclusion of a hierarchical structure, nesting the hatchery within the country, significantly improved the model fit. Actually, hatcheries located in different countries showed a different response to audit activity (Figure 3). The causes behind the heterogeneous behavior remain challenging to be understood because several underling factors could be concealed, including differences in the national legislation, cultural, social, and economic factors; structure of the productive system (i.e., several independent hatcheries vs. one or few companies owning multiple hatcheries); and so on (Baráth and Fertő, 2017, Luh, 2017). In addition, different inspectors were responsible for the audits and questionnaire administration in each county. Although the standardized nature of the selected approach should minimize this bias, a certain effect of the interviewer can not be excluded (Bowling, 2005).

The present study demonstrates the benefits of the objective evaluation of hatchery performances through a standardized questionnaire, followed by a discussion of the major required actions. The development of informatics applications could significantly facilitate this process by allowing an easier data collection and storage, fundamental for real-time analysis of the hatchery over time, and with respect to other productive scenarios. The widespread application of this approach could lead to a significant improvement in vaccine administration performances, with direct consequences on infectious disease occurrence and animal performances and indirectly on therapeutic and control-related costs. Further analysis needs to be performed to assess the relation between quality of hatchery vaccination processes and the performance of the vaccinated chickens in the field.
